# Retrograde Activation of the Extrinsic Apoptotic Pathway in Spinal-Projecting Neurons after a Complete Spinal Cord Injury in Lampreys

**DOI:** 10.1155/2017/5953674

**Published:** 2017-11-19

**Authors:** Antón Barreiro-Iglesias, Daniel Sobrido-Cameán, Michael I. Shifman

**Affiliations:** ^1^Department of Functional Biology, CIBUS, Faculty of Biology, Universidade de Santiago de Compostela, 15782 Santiago de Compostela, Spain; ^2^Shriners Hospitals Pediatric Research Center (Center for Neural Repair and Rehabilitation), Temple University School of Medicine, 3500 North Broad Street, Philadelphia, PA 19140, USA

## Abstract

Spinal cord injury (SCI) is a devastating condition that leads to permanent disability because injured axons do not regenerate across the trauma zone to reconnect to their targets. A prerequisite for axonal regeneration will be the prevention of retrograde degeneration that could lead to neuronal death. However, the specific molecular mechanisms of axotomy-induced degeneration of spinal-projecting neurons have not been elucidated yet. In lampreys, SCI induces the apoptotic death of identifiable descending neurons that are “bad regenerators/poor survivors” after SCI. Here, we investigated the apoptotic process activated in identifiable descending neurons of lampreys after SCI. For this, we studied caspase activation by using fluorochrome-labeled inhibitors of caspases, the degeneration of spinal-projecting neurons using Fluro-Jade C staining, and the involvement of the intrinsic apoptotic pathway by means of cytochrome c and V*α* double immunofluorescence. Our results provide evidence that, after SCI, bad-regenerating spinal cord-projecting neurons slowly degenerate and that the extrinsic pathway of apoptosis is involved in this process. Experiments using the microtubule stabilizer Taxol showed that caspase-8 signaling is retrogradely transported by microtubules from the site of axotomy to the neuronal soma. Preventing the activation of this process could be an important therapeutic approach after SCI in mammals.

## 1. Introduction

In humans, as in the other mammals, spinal cord injury (SCI) causes permanent disability. In mammals, one of the main reasons for the failure of recovery is caused by the inability of axotomized axons to regenerate across the injury site to reconnect to their targets. An important goal of current research aiming to develop a therapy for SCI is to promote the regeneration of damaged axons [1]. An important prerequisite for axonal regeneration would be the prevention of retrograde degeneration that could cause neuronal death or atrophy impeding the activation of axonal regrowth. Several types of central nervous system (CNS) neurons die after they suffer axonal damage. For example, retinal ganglion cells [2–4] and motoneurons [5, 6] die after axotomy. However, there is still some controversy whether spinal cord (SC) projecting neurons of the brain of mammals die after SCI. Several studies have shown the death of at least some brain neurons after SCI (opossum [7]; rats [8–12]; humans [13]). However, in two recent studies using rats, no evidence was found for the death of corticospinal neurons after SCI [14] and suggested that these neurons suffer atrophy after SCI but do not die [15]. The death or degeneration (atrophy) of descending neurons following SCI seems to involve an apoptotic process. This is suggested by the appearance of TUNEL staining and activated caspase-3 immunoreactivity in descending neurons of the brain (pontine reticular neurons [10]; corticospinal neurons [9, 11]). But, more work is needed to fully understand the molecular mechanisms that control the degeneration of descending neurons of the brain following a traumatic SCI.

In contrast to mammals, lampreys recover normal appearing locomotion spontaneously several weeks after a complete SCI [16]. This occurs due to the occurrence of plastic changes [17–19] and regenerative processes [20–22] in spinal circuits after the injury. Descending neurons of lampreys are able to regenerate their axons after a complete SC transection [23, 24] and a large percentage of spinal axons regenerate in their correct paths [16, 25]. Moreover, regenerated descending axons are able to form new functional synapsis with neurons below the site of injury [25–27]. Among the reticulospinal neurons of the lamprey brain, there are 36 identifiable giant descending neurons [23], whose axons project almost the entire length of the SC. These include the Mauthner neurons, which have crossed descending axons, and several pairs of Müller cells, which have ipsilateral descending axons. Interestingly, these identifiable descending neurons vary greatly in their regenerative abilities following SCI [23]. Some of these neurons are considered “good regenerators” (i.e., they regenerate their axon more than 55% of the times) and others are considered “bad regenerators” (i.e., they regenerate their axon less than 30% of the times). Thus, lampreys offer a model where there is an opportunity to study both enhancement and inhibition of regeneration in the same preparation. An additional advantage of the sea lamprey model of SCI is that the identifiable descending neurons and their descending axons can be visualized* in vivo* and in CNS whole-mounts due to the transparency of the lamprey brain. This allows to study the intrinsic molecular processes that determine the fate of axotomized neurons following SCI. For example, recent studies have shown that the differential expression of axonal guidance receptors in good or bad regenerators can explain, at least in part, their different regenerative abilities [22].

We and others have recently reported that a complete SCI induces the delayed death of lamprey descending neurons that, at earlier times after injury, had been identified as bad regenerators [28, 29]. The occurrence of cell death was determined based on the disappearance of Nissl staining, the loss of neurofilament expression, the absence of labeling when using retrograde tracers [28], and the early staining of these neurons with Fluoro-Jade® C (FJC) [29], which is a marker for degenerating neurons. In addition, the appearance of TUNEL staining [28] and activated caspases [30–32] in the soma of axotomized descending neurons suggested that their death after SCI is apoptotic. A recent report by Barreiro-Iglesias and Shifman (2015) has shown that caspase activation in the cell perikarya of bad-regenerating descending neurons of lampreys is preceded by the initial activation of caspases in the axotomized axons at the lesion site within the first hours after the injury. This suggests that the degenerative process is initiated at spinal levels. These data indicate that the identifiable descending neurons known to be “bad regenerators” (i.e., the M1, M2, M3, I1, I2, Mth, B1, B3, and B4 descending neurons) suffer a process of slow and delayed degeneration after SCI and, therefore, are also “poor survivors.” Thus, lampreys are a convenient vertebrate model for the* in vivo* study of molecular mechanisms underlying the death or degeneration of descending neurons after SCI.

Apoptosis is a process that occurs normally during development and aging and as a homeostatic mechanism to maintain cell populations in tissues but that can be also activated after cell damage [33]. In apoptosis, caspases are responsible for proteolytic cleavages that lead to cell disassembly (effector caspases) or that are involved in regulatory events (initiator caspases) [34]. Research indicates that there are two main apoptotic pathways: the extrinsic or death receptor pathway and the intrinsic or mitochondrial pathway. The intrinsic pathway is activated by genomic and metabolic stress, the presence of unfolded proteins, and other factors that lead to permeabilization of the outer membrane of mitochondria and the release of apoptotic proteins, mainly cytochrome c, into the cytosol. Apoptosis initiation through the intrinsic pathway usually leads to the formation of the apoptosome complex and the activation of the initiator caspase-9 [34]. The extrinsic or death receptor pathway involves the activation of death receptors. This process leads to activation of initiator caspase-8 or caspase-10 [34]. Recently, we adapted the use of fluorochrome-labeled inhibitors of caspases (FLICA) to detect the activation of caspases in whole-mount preparations of the sea lamprey brain following SCI [30]. We observed that activated caspase-8 labeling appears in the soma of “poor survivor” descending neurons within 2 weeks following a SCI [30, 31] and that the appearance of activated caspase-8 in the cell body was preceded by its activation in the injured axon rostral to the site of injury [31]. This suggests that the extrinsic pathway of apoptosis is activated in descending neurons after axotomy. In the present study, we aimed to (1) further define the activation of the extrinsic apoptotic pathway in identifiable reticulospinal neurons after SCI, (2) study the possible activation of the intrinsic apoptotic pathway, and (3) determine how the injury signals get to the cell body. Our results provide additional evidence that, after SCI, the bad-regenerating spinal-projecting neurons slowly degenerate, that only the extrinsic and not the intrinsic pathway of apoptosis is involved in this process, and that caspase-8 signaling is transported to the soma of descending neurons by microtubules.

## 2. Material and Methods

### 2.1. Animals

Wild type larval sea lampreys (*Petromyzon marinus* L.), 10–14 cm in length (4–7 years old), were obtained from streams feeding Lake Michigan (USA) or from the River Ulla (Spain) and maintained in aerated freshwater tanks at 16°C until the day of use. Experiments were approved by the Institutional Animal Care and Use Committee at Temple University and the by the Bioethics Committee at the University of Santiago de Compostela and the* Consellería do Medio Rural e do Mar* of the* Xunta de Galicia* (code JLPV/IId, Spain) and were performed in accordance with European Union and Spanish guidelines on animal care and experimentation.

### 2.2. Complete SC Transection

Before experiments, animals (*n* = 57) were deeply anesthetized by immersion in lamprey Ringer solution containing 0.1% tricaine methanesulfonate (ScienceLab, Houston, TX, USA). Complete SC transections were performed as previously described [35]. Briefly, the SC was exposed from the dorsal midline at the level of the fifth gill. Complete transection of the SC was performed with Castroviejo scissors and the transection was confirmed under the stereomicroscope. After surgery, animals were kept on ice for 1 hour allowing the wound to air dry. Each transected animal was examined 24 hours after surgery to confirm that there was no movement caudal to the lesion site. A transection was considered complete if the animal could move only its head and body rostral to the lesion. Animals were allowed to recover in aerated fresh water tanks at room temperature for different times (2 weeks, 4 weeks, 6 weeks, 10 weeks, or 4 months).

### 2.3. FJC Staining in Whole-Mounted Brain Preparations

Fluoro-Jade C is a polyanionic fluorescein derivative and is commonly used in neuroscience to stain degenerating neurons in the central nervous system, regardless of specific insult or mechanism of cell death [36, 37]. In the present study, FJC staining was used to further confirm that spinal-projecting neurons of lampreys slowly degenerate after spinal cord transection as shown previously with other markers of cell death and degeneration [28].

Brains of control animals (*n* = 3) without a complete SC transection (unlesioned) and brains of animals 4 months (*n* = 5) after the SC transection were removed in ice-cold Ringer and the dorsal choroid plexus covering the 4th ventricle was removed. The posterior and cerebrotectal commissures of the brain were cut along the dorsal midline, and the alar plates were deflected laterally and pinned flat to a small strip of Sylgard (Dow Corning Co., USA). Brains were fixed in 4% paraformaldehyde (PFA) in phosphate buffered saline (PBS) for 2 hours at room temperature and washed on a nutator for 2 hours in PBS containing 2% Tween 20. Then, brains were immersed in 80% ethanol solution containing 1% NaOH for 5 minutes, then transferred to 70% ethanol solution for 2 minutes, and rinsed in water for 2 minutes. The brains were incubated in 0.06% potassium permanganate solution for 10 min and rinsed in water for 2 minutes. The proper dilution was made by making a 0.01% stock solution of FJC dye (Chemicon, Temecula, CA, USA) in distilled water and then adding 1 mL of the stock solution to 99 mL of 0.1% acetic acid. The brains were immersed in the staining solution for 25 minutes and rinsed in water 3 times for 1 minute each. After washes, brains were mounted on Superfrost Plus glass slides (Fisher Scientific, MA, USA) and coverslipped using Prolong (Invitrogen, USA) as an antifade reagent. Control brains were processed in parallel with brains of animals 4 months after injury.

### 2.4. Detection of Activated Caspases in Whole-Mounted Brain Preparations

The Image-iT LIVE Green Poly Caspases Detection Kit (Cat. number I35104, Invitrogen, USA), Image-iT LIVE Green Caspase-3 and -7 Detection Kit (Cat. number I35106, Invitrogen), and the Image-iT LIVE Green Caspase-8 Detection Kit (Cat. number I35105, Invitrogen) were used to detect activated caspases in identified reticulospinal neurons of larval sea lampreys after a complete SC transection. These kits contain 1 vial (component A of the kit) of the lyophilized FLICA reagent (FAM-VAD-FMK for the detection of all activated caspases, FAM-DEVD-FMK for the detection of activated caspase-3 and caspase-7, and FAM-LETD-FMK for the specific detection of activated caspase-8). Experiments were done as previously described [30, 31]. Experiments for the detection of all activated caspases were performed in animals 2 weeks after the complete SC transection (*n* = 5). Experiments for the detection of activated caspase-8 were in animals 4 weeks (*n* = 4) after the complete SC transection. Experiments for detection of activated caspase-3 and caspase-7 were done in control unlesioned animals (*n* = 5) and in animals 6 weeks (*n* = 3) and 10 weeks (*n* = 4) after the complete SC transection.

### 2.5. Double Cytochrome c and Complex V*α* Immunofluorescence in Whole-Mounted Brain Preparations

Brains of control unlesioned animals (*n* = 4), and animals 2 weeks (*n* = 5) and 4 weeks (*n* = 5) after the complete SC transection were dissected, fixed, and processed as for the FJC staining (see above). After PFA fixation, brains were washed on a nutator at room temperature twice (1 hour each wash) in PBS containing 2% Tween 20 and then twice (30 minutes each wash) in maleate buffer containing 0.2% Tween 20. The brains were immersed for 1 hour in maleate buffer containing 15% normal goat serum (MitoSciences, Eugene, Oregon, USA) and incubated in the same solution containing a mouse anti-cytochrome c monoclonal IgG2a antibody (1 : 500; clone 37BA11; catalog number MSA07; MitoSciences) and a mouse anti-complex V*α* (ATP synthase subunit alpha) monoclonal IgG2b antibody (1 : 500; clone 15H4C4; Cat. number MSA07; MitoSciences) for 2 days at 4°C. After washes as above, the brains were incubated in the same solution containing a goat anti-mouse IgG2a antibody conjugated to fluorescein isothiocyanate (FITC) (1 : 500; Cat. number MSA07; MitoSciences) and a goat anti-mouse IgG2b antibody conjugated to Texas Red (1 : 500; Cat. number MSA07; MitoSciences) overnight at 4°C. Then, the brains were rinsed in PBS containing 0.1% Tween 20, mounted on Superfrost Plus glass slides (Fisher Scientific, MA, USA) and coverslipped using Prolong (Invitrogen) as an antifade reagent.

The specificity of the anti-cytochrome c antibody was tested by Western blot using larval sea lamprey total protein from combined spinal cord and brain samples homogenates (*n* = 2). For extract preparation, the sample was lysed in TNN buffer (50 mM Tris, 150 mM NaCl, and 0.5 NP-40) with protease inhibitor cocktail (Sigma). The protein concentration was measured by the Bradford assay: 30 mg lysate was loaded per well for SDS-PAGE and proteins were transferred onto PVDF or nitrocellulose membranes. Western analysis was conducted by incubation with the anti-cytochrome c antibody (1 : 1000) overnight at 4°C, followed by incubation with HRP-tagged secondary anti-mouse antibody (1 : 5000; 1 h at room temperature; Americana Qualex, San Clemente, CA, USA). Proteins were visualized using ECL (GE Health Sciences, Piscataway, NJ, USA). The antibody recognized a single band of about 13 kDa (Figure 5(d)), which corresponds to the molecular size of cytochrome c of mammalian species. Colocalization of cytochrome c and complex V*α* immunoreactivities in neurons of normal animals (see Results) further confirmed the specificity of both antibodies and the validity of the monoclonal anti-complex V*α* antibody as a mitochondrial marker. As a control for secondary antibodies, the incubation with primary antibodies was omitted. No immunoreactivity was observed in these experiments.

### 2.6. Taxol (Placlitaxel) Treatment

Immediately after the complete SC transection, a small piece of Gelfoam soaked with 10 *μ*L of either 1 mM Taxol (Molecular Probes, Eugene, OR, USA) in ethanol (*n* = 7) or 10 *μ*L of ethanol alone (*n* = 5) was placed on the top of the SC at the site of injury. Animals recovered for 1 or 2 weeks and their brains/spinal cords were processed for detection of activated caspase-8 as above. Control animals that did not receive Taxol treatment were always processed in parallel with experimental animals treated with Taxol.

### 2.7. Imaging and Preparation of Figures

Photomicrographs were taken using a Nikon Eclipse 80i microscope equipped with a CoolSNAP ES (Roper Scientific, USA) camera or with a spectral confocal microscope (model TCS-SP2; Leica, Wetzlar, Germany). Images were always taken under the same microscope conditions for control or treated animals. Quantification of mean fluorescent intensity of each identifiable neuron was done using the histogram function of the Fiji software. For the preparation of figures, brightness and contrast were minimally adjusted using Adobe Photoshop CC software and lettering was added.

### 2.8. Statistical Analyses

Statistical analysis was carried out using Prism 6 (GraphPad software, La Jolla, CA). Data were presented as mean ± SEM. Normality of the data was determined by the D'Agostino-Pearson normality test. The correlation (Pearson test) between fluorescence intensity of the FLICA labeling (present results) and the regenerative ability of the descending neurons [23] was analyzed. Differences in fluorescence intensity between control and Taxol treated animals were analyzed by means of a two-tailed paired Student's *t*-test.

## 3. Results

### 3.1. Levels of Activated Caspases 2 Weeks after a Complete SC Transection Correlate Significantly with the Long-Term Regenerative Ability of Identifiable Neurons

In previous studies [23, 38], some reticulospinal neurons of the sea lamprey were identified as “bad regenerators” (M1, M2, M3, I1, I2, Mth, B1, B2, B3, and B4) because their axons had a low probability of regenerating after axotomy due to a complete SC transection. In later studies, almost the same cells were identified as “poor survivor” neurons because they have a high likelihood of degenerating and dying after axotomy (the M1, M2, M3, I1, I2, Mth, B1, B3, and B4 neurons) [28].

**Figure 1:**
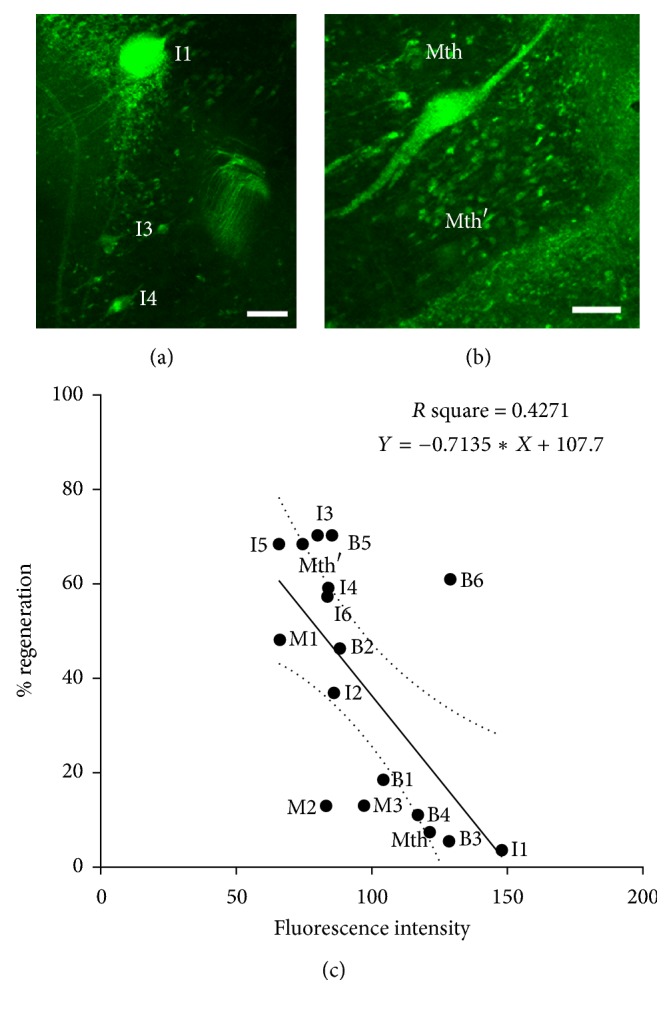
Confocal photomicrographs of dorsal views of whole-mounted brains of larval sea lampreys showing FAM-VAD-FMK labeling in axotomized identified spinal-projecting neurons 2 weeks after SCI. (a) I1, I3, and I4 neurons. (b) Mth and Mth′ neurons. Note that fluorescence intensity in I1 and Mth neurons is higher than I3, I4, and Mth′ neurons. Rostral is to the top and the ventricle is to right in all figures. (c) Linear regression analysis shows the inverse correlation between the regenerative ability of identified SC projecting neurons and their fluorescence intensity reflecting caspase activation (95% confidence intervals for slope −1.168 to −0,2588; Sy.x = 20.50; *p* = 0.0044). The dotted lines indicate the 95% confidence interval. Identity of RS neurons is shown adjacent to their corresponding data points. I1, I3, and I4: Müller cells of the isthmic region; Mth: Mauthner cell; Mth′: auxiliary Mauthner cell. Scale bars: 50 *μ*m.

Also, previous studies revealed that bad regenerator/poor survivor neurons show high levels of activated caspases in the first 2 weeks following a complete SC transection as revealed by FLICA labeling [30–32] and that intense FLICA labeling and TUNEL staining are observed in bad regenerators several weeks after a complete SC transection [32]. However, these studies never established a statistical correlation between the intensity of FLICA labeling in the first 2 weeks after the injury and the long-term regenerative ability of the individually identifiable neurons. Here, we used a poly-caspase FLICA reagent (FAM-VAD-FMK) to detect all activated caspases 2 weeks following a complete SC transection (Figures 1(a) and 1(b)) and correlated the levels of activated caspases in identifiable neurons with their known regenerative ability (% of times in which a specific neuron regenerates its axon across the lesion site; based on the results of [23]). This revealed a significant correlation (*p* = 0.0044; Pearson test) between the level of activated caspases (fluorescence intensity) and the known regenerative ability of the identifiable neurons (Figure 1(c)).

### 3.2. Long-Term Detection of Specific Activated Caspases in Bad Regenerators

**Figure 2:**
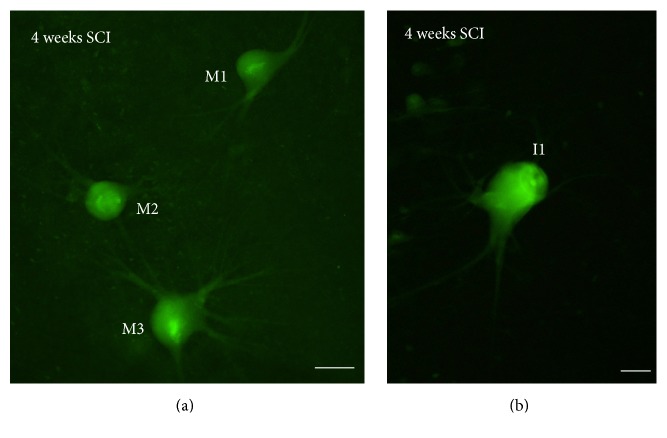
Photomicrographs of dorsal views of whole-mounted brains of larval sea lampreys showing FAM-LETD-FMK labeling in axotomized descending neurons 4 weeks after SCI ((a) and (b)). Rostral is to the top and the ventricle is to left in all figures. I1: Müller cell of the isthmic region; M1–M3: Müller cells 1 to 4. Scale bars: 50 *μ*m in (a) and 20 *μ*m in (b).

In previous studies, we reported intense FLICA labeling 2 weeks after lesion in bad regenerators using the FLICA reagent specific for the detection of activated caspase-8 (FAM-LETD-FMK) [30]. Here, we extended our analyses and observed that at, one month posttransection, FAM-LETD-FMK labeling is still observed in “poor survivor” SC projecting neurons of the brainstem (M1, M2, M3, I1, I2, Mth, B1, B3, and B4; Figure 2).

**Figure 3:**
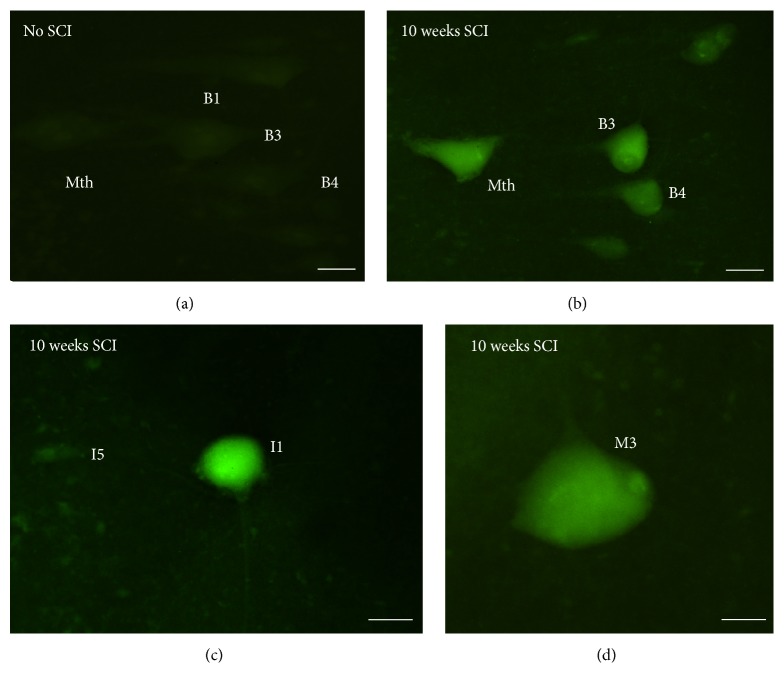
Photomicrographs of dorsal views of the whole-mounted brain of larval sea lampreys showing the presence of intense FAM-DEVD-FMK labeling in “poor survivor” spinal-projecting neurons 10 weeks after a complete spinal cord transection ((b)–(d)) as compared to controls without injury (a). Note the absence of labeling in an I5 cell in (c). Rostral is to the top and the ventricle to the left in all figures. B1, B3, and B4: Müller cells of the bulbar region; I1 and I5: Müller cells of the isthmic region; M3: Müller cell 3; Mth: Mauthner cell. Scale bars: 50 *μ*m in (a)–(c) and 15 *μ*m in (d).

Incubation of the brains with the FLICA reagent FAM-DEVD-FMK revealed the presence of activated caspase-3 or caspase-7 in identified SC projecting brainstem neurons at 10 weeks posttransection (Figure 3), but not in neurons of untransected control animals (Figure 3(a)). Little or no labeling was observed 6 weeks posttransection (not shown). Intense FAM-DEVD-FMK labeling was observed in the cell bodies of identified spinal-projecting neurons known to be “poor survivors” (the M1, M2, M3, I1, I2, Mth, B1, B3, and B4 neurons). Interestingly, the morphology of these neurons differed from that of the same neurons in control animals. The neurons seemed to have suffered atrophy and had fewer dendrites than normal neurons (Figure 3(b)–3(d)). The delay between the detection of activated caspase-8 and the detection of activated caspase-3/caspase-7 may account for the slow process of cell death observed in these neurons after axotomy [28].

### 3.3. Staining with Fluoro-Jade C Is Consistent with Protracted Degeneration of Axotomized Spinal-Projecting Neurons

**Figure 4:**
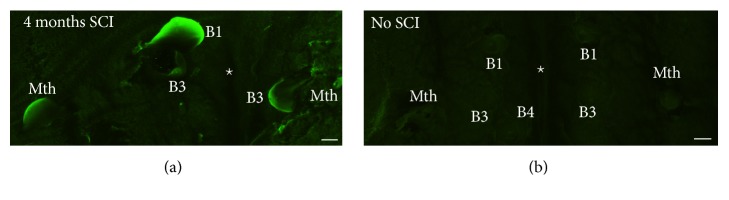
Photomicrographs of dorsal views of whole-mounted brains of larval sea lampreys showing Fluoro-Jade C staining in swollen reticulospinal neurons 4 months after a complete SC transection (a). Note the absence of labeling in control animals without SC transection (b). The star indicates the location of the ventricle. Rostral is to the top in the figures. B1, B3, and B4: Müller cells of the bulbar region; Mth: Mauthner cell. Scale bars: 12.5 *μ*m.

FJC staining (Figure 4) was not observed in identifiable spinal-projecting neurons of control unlesioned animals (Figure 4(b)). Four months after the SC transection, intense FJC labeling was observed in swollen identifiable reticulospinal neurons previously identified as “bad regenerators” (M1, M2, M3, I1, I2, Mth, B1, B3, and B4) (Figure 4(a)). This was consistent with the results of Busch and Morgan [29] and extended the period in which FJC staining was detected to 4 months, confirming that spinal-projecting neurons of lampreys degenerate very slowly after SCI.

### 3.4. Cytochrome c Is Not Released from Mitochondria of Spinal-Projecting Neurons after the Complete SC Transection

The presence of activated caspase-8 in descending neurons 2 [30] and 4 (see above) weeks after a complete SC transection suggests that the extrinsic apoptotic pathway is activated in descending neurons after axotomy. To determine whether the intrinsic pathway is also involved in the death of these neurons after axotomy, we performed double immunofluorescence experiments in whole-mounted brain preparations, using anti-cytochrome c and anti-complex V*α* antibodies. Cytochrome c release from mitochondria is a key step in the development of the intrinsic apoptotic pathway because it allows the formation of the apoptosome complex and subsequent activation of caspase-9 (see above).

**Figure 5:**
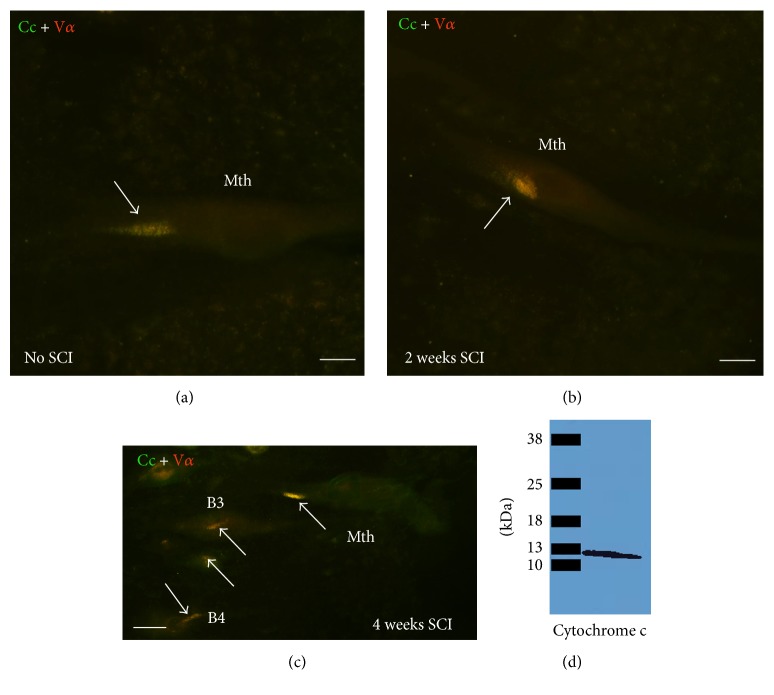
Photomicrographs of dorsal views of whole-mounted brains of larval sea lampreys showing colocalization of cytochrome c and complex V*α* immunoreactivities (arrows) in identifiable reticulospinal neurons of control animals (a) and animals 2 (b) and 4 (c) weeks after a complete SCI. Rostral is to the top and the ventricle to the left in all figures. (d) Western blot showing that the anti-cytochrome c antibody recognizes a single band of the expected molecular weight for cytochrome c (about 13 kDa). B3 and B4: Müller cells of the bulbar region; Cc: cytochrome c; Mth: Mauthner cell. Scale bars: 20 *μ*m in (a) and (b) and 15 *μ*m in (c).

In immunofluorescence experiments, colocalization of cytochrome c and complex V*α* immunoreactivities was observed as a cluster of punctuate staining in the cell bodies of all identifiable spinal-projecting neurons in control unlesioned animals (Figure 5(a)) and in animals 2 and 4 weeks posttransection (Figures 5(b) and 5(c)). This shows that at time points after SCI in which activated caspase-8 is already detected in the cell bodies of “poor survivor” neurons ([30]; present results), cytochrome c has not been released from the mitochondria.

### 3.5. Taxol Treatment Prevents Caspase-8 Retrograde Activation in Spinal-Projecting Neurons after SCI

**Figure 6:**
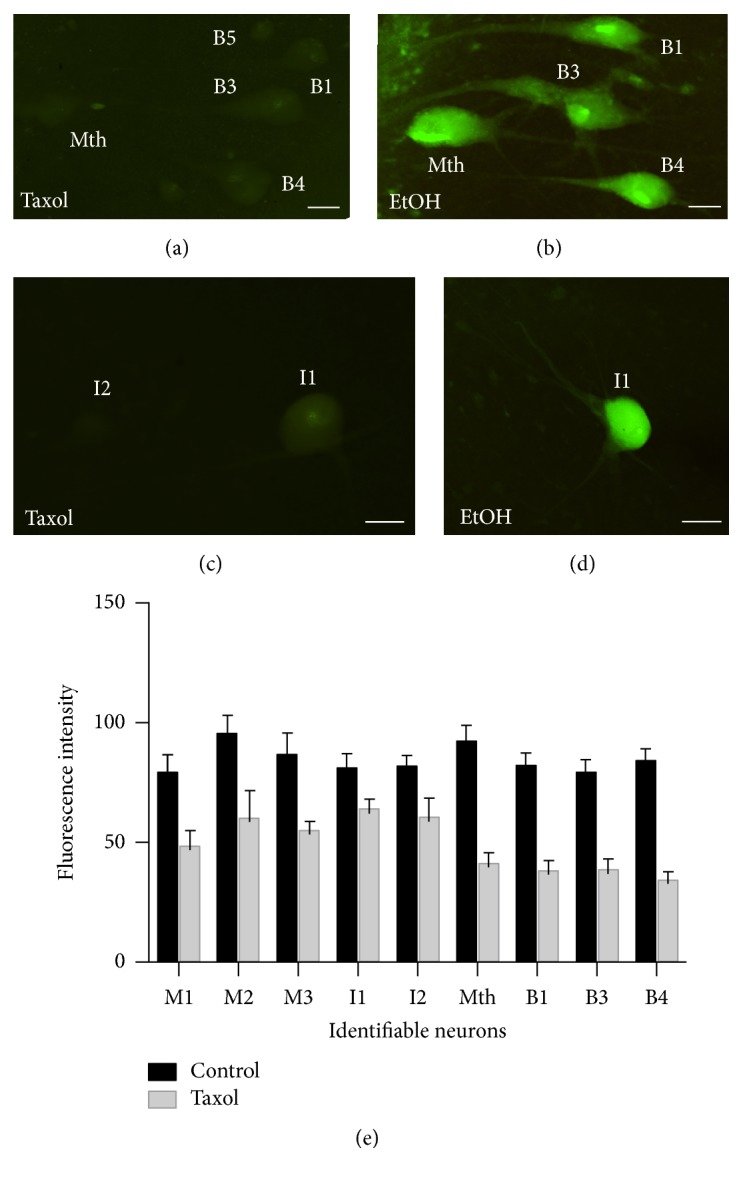
Photomicrographs of dorsal views of whole-mounted brains of larval sea lampreys showing reduced FAM-LETD-FMK labeling in identifiable “poor survivor” SC projecting neurons after Taxol ((a) and (c)) treatment as compared with control animals treated with ethanol alone ((b) and (d)). (e) Graph revealing a significant difference (*p* < 0.0001; two-tailed paired Student's *t*-test) in fluorescence intensity of the FAM-LETD-FMK labeling in identifiable neurons of control and Taxol treated animals. Rostral is to the top in all figures. The ventricle is to the right in all figures. B1 and B3–B5: Müller cells of the bulbar region; I1 and I2: Müller cells of the isthmic region; Mth: Mauthner cell. Scale bars: 50 *μ*m in (a), (c), and (d) and 25 *μ*m in (b).

The initial activation of caspase-8 at the site of injury, followed by its progressively proximal appearance toward the cell bodies of “poor survivor” SC projecting neurons suggests that either the signal/s that activate caspase-8 and/or activated caspase-8 itself is retrogradely transported from the site of axotomy to the cell bodies [31]. To determine whether the centripetal movement of caspase-8 activation depends on microtubule-based retrograde transport from the site of axotomy, we applied the microtubule stabilizer Taxol (placlitaxel) to the SC at the site of a complete transection. Pieces of Gelfoam soaked either with ethanol (control) or with a solution of 1 mM Taxol in ethanol were inserted into the lesion site. Brains from these animals were analyzed 2 weeks later for the presence of activated caspase-8 as above (Figure 6). Intense FAM-LETD-FMK labeling was observed in “poor survivor” spinal-projecting neurons of control animals (Figures 6(b) and 6(d)), but, in animals treated with Taxol, these neurons were either unlabeled or exhibited only very weak labeling (Figures 6(a) and 6(b)). Statistical analyses revealed a significant difference in caspase-8 activation (fluorescence intensity) between control and Taxol treated animals (*p* < 0.0001; two-tailed paired Student's *t*-test; Figure 6(e)).

**Figure 7:**
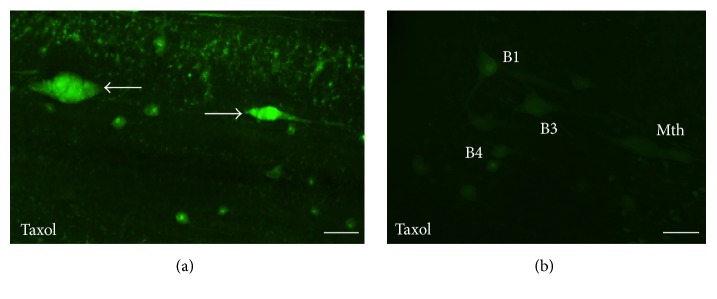
Photomicrographs of dorsal views of the whole-mounted spinal cord (a) and brain (b) of a larval sea lamprey treated with Taxol showing the presence of FAM-LETD-FMK labeling in the tip of descending axons 1 week after treatment (arrows in (a)) and the absence of labeling in identifiable descending neurons of the brain (b). Rostral is to the right in (a) and to the top in (b). The ventricle is to the left in (b). B1, B3, and B4: Müller cells of the bulbar region; Mth: Mauthner cell. Scale bars: 40 *μ*m in (a) and 50 *μ*m in (b).

To determine whether the Taxol treatment caused inhibition of caspase-8 activation itself or inhibited microtubule-based retrograde transport of caspase-8 activity from the site of axotomy, some animals were studied 1 week posttransection (Figure 7). As in the animals studied 2 weeks after injury, no labeling was observed in spinal-projecting neurons (Figure 7(b)). However, labeling was observed in the descending axons close to the site of injury (Figure 7(a)). This indicates that the Taxol treatment inhibited the transport of the signal/s that activate caspase-8 or the transport of activated caspase-8 itself but did not prevent the initial process of caspase-8 activation.

## 4. Discussion

Present findings, together with previous results [28–32], show that, in lampreys, axotomy due to SCI consistently causes retrograde degeneration and apoptotic death in individually identified spinal cord-projecting neuron; that is, they are “poor survivors,” and that these are the same cells that are “bad regenerators” [23]. The persistence of FJC staining 4 months after the injury, together with the late detection of activated caspase-3/caspase-7 (present results) and TUNEL staining [28] in these neurons prior to their disappearance, indicates that axotomy in the SC causes the activation of a very slow process of cell death in SC projecting neurons. The presence of activated caspase-8 and the lack of cytochrome c release indicate that the extrinsic apoptotic pathway is the main mediator of this cell death process after axotomy. Our results also suggest that the centripetal activation of caspases depends on microtubule-based retrograde transport of stress signals.

Studies in mammals have shown that a proportion of SC projecting neurons die or undergo atrophy after damage to their axon at the spinal level and that this may depend on activation of an apoptotic process (see Introduction). Clearly, prevention of this process would be a prerequisite for designing therapies to promote regeneration of descending pathways after SCI [39]. Therefore, it is of great importance to understand the molecular processes that lead to the retrograde degeneration of injured descending pathways after SCI. This could also inform research in other types of CNS injuries like optic nerve injuries or stroke, where apoptotic processes are also activated [40–45]. Previous results in lampreys showed that axotomy at the spinal level initially leads to activation of caspase-8 in the injured axon at the site of injury and subsequently in the cell body of descending neurons [30]. This is in agreement with reports showing that axotomy induces caspase-8 activation in retinal ganglion [44, 45] and olfactory receptor [46] neurons. Other studies have also revealed the importance of caspase-2 and caspase-6 in causing the death of retinal ganglion cells following optic nerve transections [41–43]. Experiments using the microtubule stabilizer Taxol show that, both in SC projecting neurons (present results) and in olfactory neurons [46], the retrograde activation of caspase-8 in the perikaryon after axotomy depends on microtubule-based retrograde transport from the injured axon. In olfactory neurons, the appearance of activated caspase-8 in the cell body depended on the microtubule-based retrograde transport of activated caspase-8, probably by its association with dynactin p150^Glued^ [46]. We cannot rule out the possibility that other retrograde signals may promote activation of local procaspase-8 in the cell body and along the axon of SC projecting neurons. As in olfactory neurons [46], axotomy of SC projecting neurons in lampreys caused sequential activation of caspase-8 and caspase-3 or caspase-7 (present results). Our results indicate that Taxol, or other microtubule stabilizers like epothilone B, may be a beneficial therapeutic option after SCI not only to promote axonal regeneration, as previously reported for axons in the mammalian SC [47, 48] and optic nerve [49], but also to avoid the retrograde degeneration of SC projecting neurons by preventing the retrograde transport of caspase-8 or other stress signals. Of note, the retrograde activation of neuronal degeneration might be slow in lampreys due to slower microtubule dynamics that occur in cold-living fishes [50, 51].

Studies in mammals also reported increased levels of activated caspase-8 in the SC after injury [52–55]. Because these analyses used immunoblots of SC homogenates [52–55], it is not known if the activation of caspase-8 occurred in intraspinal cells, in descending axons or in both. Neutralization of the proapoptotic cytokines tumor necrosis factor-related apoptosis-inducing ligand [55] or tumor necrosis factor-alpha [55] promoted a decrease in caspase-8 activation after SCI. Dependence receptors could also be important players in the initiation of apoptotic processes after CNS damage. Dependence receptors, like Neogenin or UNC5 receptors, trigger extrinsic apoptotic processes in the absence of their ligands [56, 57]. Recent work has shown that an inhibition of Neogenin localization in lipid rafts promotes survival and axonal growth after SC or optic nerve injuries [58]. Interestingly, recent work in lampreys has also shown that knock down of Neogenin in descending neurons of lampreys promotes axonal regeneration [22]. Another study in lampreys has also shown that knockdown of the small GTPase RhoA enhances axon regeneration and inhibits retrograde apoptosis following SCI [59]. The results of our experiments in lampreys could also be translated to mammalian injured descending axons because mechanisms of caspase-activated apoptosis are evolutionary conserved [60, 61]. The sea lamprey could be an interesting* in vivo* animal model to study the role of proapoptotic cytokines, dependence receptors, or other molecules like RhoA in the intra-axonal activation of caspase-8 after SCI.

## Abbreviations Used in the Figures

B1–B6: Müller cells of the bulbar region (middle rhombencephalic reticular nucleus)
Cc: Cytochrome c
EtOh: Ethanol
I1–I6: Müller cells of the isthmic region
M1–M4: Müller cells 1 to 4
Mth: Mauthner cell
Mth′: Auxiliary Mauthner cell
SCI: Spinal cord injury.
